# “It’s very complicated”: a qualitative study of medicines management in intermediate care facilities in Northern Ireland

**DOI:** 10.1186/s12913-015-0869-1

**Published:** 2015-06-02

**Authors:** Anna N. Millar, Carmel M. Hughes, Cristín Ryan

**Affiliations:** School of Pharmacy, Queen’s University Belfast, 97 Lisburn Road, Belfast BT9 7BL, Northern Island UK; Primary Care Pharmacy, Queen’s University Belfast, 97 Lisburn Road, Belfast, BT9 7BL Northern Island UK; Pharmaceutical Science and Practice, Queen’s University Belfast, 97 Lisburn Road, Belfast, BT9 7BL Northern Island UK

**Keywords:** Intermediate care, Medicines management, Pharmacy, Older people, Qualitative

## Abstract

**Background:**

Intermediate care (IC) describes a range of services targeted at older people, aimed at preventing unnecessary hospitalisation, promoting faster recovery from illness and maximising independence. Older people are at increased risk of medication-related adverse events, but little is known about the provision of medicines management services in IC facilities. This study aimed to describe the current provision of medicines management services in IC facilities in Northern Ireland (NI) and to explore healthcare workers’ (HCWs) and patients’ views of, and attitudes towards these services and the IC concept.

**Methods:**

Semi-structured interviews were conducted, recorded, transcribed verbatim and analysed using a constant comparative approach with HCWs and patients from IC facilities in NI.

**Results:**

Interviews were conducted with 25 HCWs and 18 patients from 12 IC facilities in NI. Three themes were identified: ‘concept and reality’, ‘setting and supply’ and ‘responsibility and review’. A mismatch between the concept of IC and the reality was evident. The IC facility setting dictated prescribing responsibilities and the supply of medicines, presenting challenges for HCWs. A lack of a standardised approach to responsibility for the provision of medicines management services including clinical review was identified. Whilst pharmacists were not considered part of the multidisciplinary team, most HCWs recognised a need for their input. Medicines management was not a concern for the majority of IC patients.

**Conclusions:**

Medicines management services are not integral to IC and medicine-related challenges are frequently encountered. Integration of pharmacists into the multidisciplinary team could potentially improve medicines management in IC.

## Background

Intermediate care (IC) is a care setting that has evolved in response to the ageing population, the increasing pressure faced by acute healthcare services and the resulting need for alternatives to hospital-based care. Although the term ‘IC’ originated in the United Kingdom (UK), other countries have adopted similar strategies; several equivalent healthcare models are used globally and are denoted by a variety of terminologies including ‘sub-acute care’, ‘post-acute care’ and ‘transition care’ [1].

IC is broadly defined in the UK as ‘*a range of integrated services to prevent unnecessary hospital admission*, *promote faster recovery from illness*, *support timely discharge and maximise independent living*’ [2]. IC services may be provided either in IC facilities or in the patient’s own home. The types of individuals receiving IC and the key principles of its provision are detailed in Table 1.

**Table 1:** Overview of intermediate care, Department of Health Social Services and Public Safety, Northern Ireland [39]

• Intermediate care services should be targeted at people who would otherwise face:
– Inappropriate admission to acute in-patient care;
– Long-term residential/nursing home care;
– Unnecessary prolonged hospital stays; or
– Continuing in-patient care.
Key Principles:
• IC should be provided on the basis of a comprehensive person-centred assessment of need, resulting in a structured individual care plan that, where appropriate, involves active therapy, treatment or opportunity for recovery;
• IC should have a planned outcome of maximising independence and typically enabling service users to remain or resume living at home;
• IC should be time-limited, usually no longer than six weeks and frequently as little as 1-2 weeks or less; and
• IC should involve cross-professional working, with a single assessment framework, increasingly integrated professional records and shared protocols.

An integral component of IC is multidisciplinary team involvement to meet patients’ care needs [2]. This approach has been credited with improving continuity of patient care during the transition between healthcare settings [3]. However, the role of pharmacists within IC has neither been defined nor evaluated, despite recognition that pharmacists’ skills may be of value in IC [4].

IC services are targeted towards an older population for whom prescribed medicines comprise a fundamental component of their care. Older patients are more likely to have multiple morbidities for which they are prescribed numerous medications, increasing their risk of experiencing adverse drug events (ADEs) [5]. Additionally, up to 30 % of hospital admissions in older adults are thought to be ADE-related [6]. Such ADEs, the majority of which are thought to be avoidable, are associated with considerable morbidity, mortality and increased healthcare costs [7]. Furthermore, the introduction of IC as a setting between primary and secondary care has created an additional healthcare interface across which medicines have to be managed. Receiving care in numerous settings puts individuals at increased risk of discontinuity of care in relation to medicines management [8]. Due to these multiple factors, intrinsic to the IC setting, it is therefore imperative that medicines management is embedded within such services.

This study aimed to describe the current provision of medicines management services in IC facilities in NI and to explore healthcare workers’ (HCWs) and patients’ views of and attitudes towards this service along with the overall concept of IC as a care setting. In addition, this study sought to identify HCWs’ perceived barriers and facilitators to the provision of medicines management services.

## Methods

### Study design and recruitment

A qualitative study design, involving semi-structured interviews, was adopted. Three of the five local public authorities that manage and deliver health and social care in NI (hereafter referred to as Trusts) were included. Only those IC services that were provided in facilities were included; thus those IC services provided to patients in their own homes were not included. Of the Trusts not included, one had no identifiable IC facility(s) at the time of the study and there was on-going research in IC in the second Trust. IC facilities with ≥5 beds in the three Trusts involved were identified (n = 12), facility managers were contacted by the researcher (AM), and all agreed to participate. HCWs, defined as those with direct involvement with medicines management within the facility were eligible to participate. HCWs were recruited by letters distributed to all eligible staff working within the participating IC facilities. Patient participants were recruited via nominated HCWs in each facility. The inclusion criteria stipulated that at the time of the interview, patient participants were aged >18 years, present in the IC facility for a minimum of two weeks, taking four or more regular medications and did not have cognitive or communicative impairment.

### Interview schedules

**Fig. 1:**
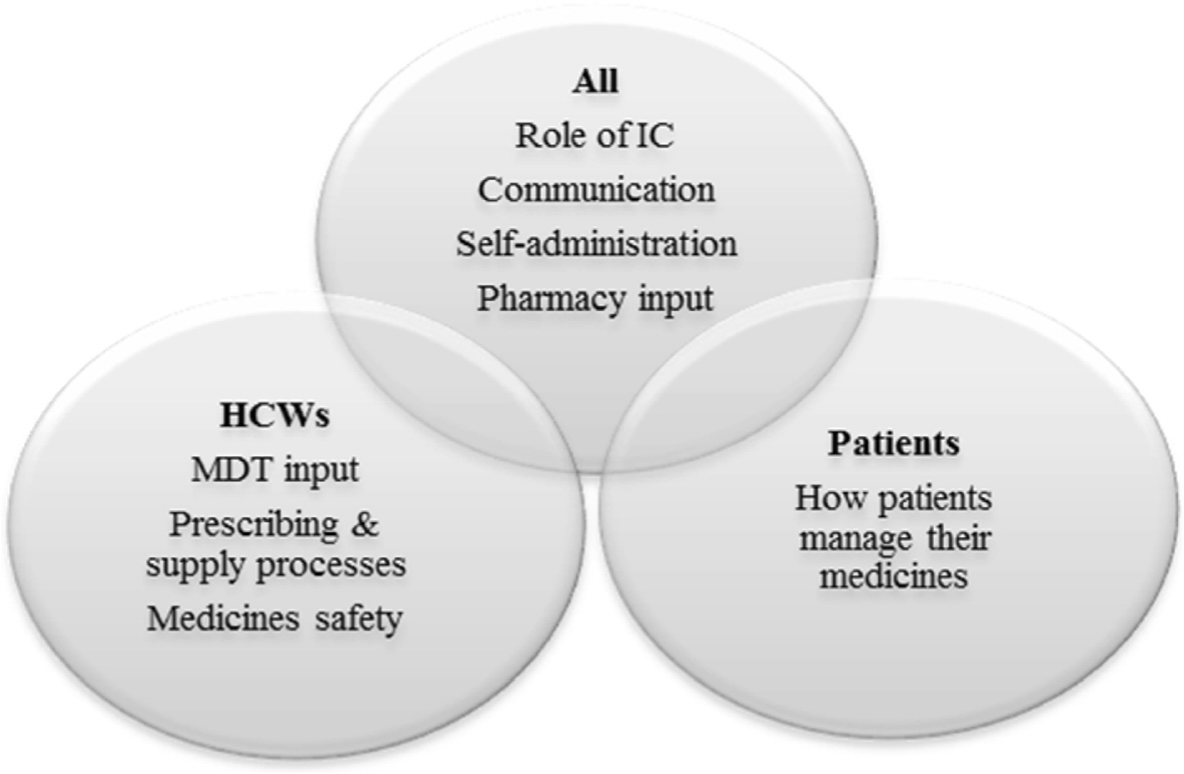
Interview schedule content (HCWs = healthcare workers; MDT = multi-disciplinary team)

A series of semi-structured interview schedules were developed, the content of which was informed by a literature review of the subject area^1^ and discussions within the research team. The schedules differed slightly in content and language used, depending on the interview’s target group (see Fig. 1). The interview schedules were then piloted to refine the content and ensure validity.

At the interview, HCWs were provided with a hard copy of the key principles of IC (see Table 1) to aid discussion. All interviews were undertaken following written, informed consent and conducted by the researcher (AM), trained in interview techniques. As interviews progressed, emerging themes were identified and the interview schedules were amended to explore new areas of interest. Recruitment and data collection ceased when data saturation was deemed to have occurred, noted as the appearance of no new themes emerging with subsequent interviews.

### Analysis

Data were transcribed verbatim, all identifiers were removed and codes assigned to participants, and imported to NVivo® to facilitate analysis, using a constant comparative approach frequently employed in this area of research [9]. This method involves the simultaneous coding and analysis of data in order to develop and refine themes and explore their relationships to one another [10]. By including both a variety of HCWs and patients, the research question was explored from various perspectives, a concept known as data triangulation; such an approach has been credited with improving the validity of qualitative study findings [11, 12]. The initial analysis was completed by one researcher (AM), with a random sample of transcripts subjected to the same analysis by a second researcher (CR). Consensus on the emergent themes was reached by discussion among all three researchers (AM, CR, CH). Ethical approval was granted by the Office for Research Ethics Committees Northern Ireland (ORECNI) and governance approval was provided by each of the three Trusts involved.

## Results

Twelve IC facilities from three Trusts (A, B & C) were recruited. These facilities varied in their organisational characteristics, summarised in Table 2. A total of 43 participants (18 patients and 25 HCWs) were recruited over a six month period from June 2013 - November 2013. Table 3 summarizes the demographic information pertaining to all participants, who were assigned codes according to the sequence of the interview. Interviews with HCWs lasted, on average, 40 minutes, whilst patient interviews lasted 10-15 minutes. Three overarching themes were identified: ‘concept and reality’, ‘setting and supply’ and ‘responsibility and review’. A mismatch between the previously defined concept of IC and the reality of the services provided in the facilities was highlighted. The setting in which IC was delivered dictated prescribing processes and the supply of medicines, which presented a number of challenges for HCWs in the facilities. A lack of a standardised approach to the responsibility for the provision of medicines management services including the clinical review of medicines and the provision of medication counselling was also identified. Despite holding positive views of IC services, management of their medicines within IC was not an area of concern for the majority of patients. Additionally, whilst pharmacists were not considered part of the multidisciplinary team, the majority of HCWs identified a need for increased pharmacy input. These issues are described in greater detail in the following sections.

**Table 2:** Settings of IC facilities included in the study

*Nursing home facilities* (*n* = *5*)
A more recent model of IC, these privately owned nursing home facilities which, in addition to long-term nursing care, also provided IC in varying numbers of beds purchased by the Trusts. Medical care provision in these homes ranged from daily visits to ‘when required’ visits from a contracted GP (medical officer). In addition, three of the facilities had weekly input from a consultant geriatrician.
*Residential care home facilities* (*n* = *3*)
These facilities were Trust-owned residential care homes, staffed primarily by healthcare assistants (support staff who provide basic care under the guidance of qualified healthcare professionals). Of the three IC facilities in this category, two managers had nursing backgrounds and one had a social work background. There was less medical input in these facilities in comparison to the other categories and medical cover was provided by either the patients’ own GP or a local, contracted GP.
*Non*-*acute hospital facilities* (*n* = *4*)
These were long-established facilities, sometimes referred to as ‘community hospitals’, whose services pre-date the label of IC. One facility included in this category functioned very much as a typical hospital ward with medical cover provided 24/7 by Trust medical staff including junior doctors, registrars and consultants. The three other facilities in this category also had hospital ward-like environments, although medical cover was provided on a day-time basis by local GPs whose practices were within the vicinity of the facility.

**Table 3:** Demographic profile of interview participants (n = 43)

Participant^a^	Description	IC facility setting	Trust
HCW1	Home manager	Residential home	A
HCW2	Senior healthcare assistant	Residential home	A
P1	Female	Residential home	A
HCW3	Nurse	Nursing home	A
HCW4	Nurse	Nursing home	A
P2	Male	Nursing home	A
HCW5	Home manager	Nursing home	A
HCW6	Nurse	Nursing home	A
HCW7	Senior healthcare assistant	Residential home	A
HCW8	Senior healthcare assistant	Residential home	A
P3	Female	Residential home	A
P4	Male	Residential home	A
P5	Female	Residential home	A
HCW9	Home manager	Nursing home	A
HCW10	Nurse	Nursing home	A
HCW11	Nurse	Nursing home	A
P6	Female	Nursing home	A
P7	Male	Nursing home	A
HCW12	Home manager	Residential home	A
HCW13	Pharmacist	Non-acute hospital	B
HCW14	Nurse	Non-acute hospital	B
HCW15	Nurse	Non-acute hospital	B
P8	Female	Non-acute hospital	B
P9	Male	Non-acute hospital	B
HCW16	Nurse	Non-acute hospital	B
HCW17	Nurse	Non-acute hospital	B
HCW18	Nurse	Non-acute hospital	B
P10	Male	Non-acute hospital	B
P11	Female	Non-acute hospital	B
HCW19	Nurse	Non-acute hospital	B
P12	Male	Non-acute hospital	B
P13	Female	Non-acute hospital	B
HCW20	Nurse	Nursing home	C
HCW21	General Practitioner	Nursing home	C
P14	Male	Nursing home	C
P15	Female	Nursing home	C
HCW22	Nurse	Non-acute hospital	C
HCW23	Medical doctor	Non-acute hospital	C
P16	Male	Non-acute hospital	C
P17	Female	Non-acute hospital	C
P18	Male	Non-acute hospital	C
HCW24	Consultant Geriatrician	Nursing home	C
HCW25	Pharmacist	Nursing home	C

^a^Key: HCW = Healthcare worker, P = Patient

### Concept and reality

HCWs noted that the concept of IC was not being fully realised and several aspects of the reality of service contrasted with the definition of IC. At the outset, the term ‘*intermediate care*’ was not frequently used by interviewees, with most identifying the service as ‘*rehabilitation*’. Some participants commented that they avoided using the term ‘IC’ as they viewed the terminology and concept of IC to be poorly understood in the wider health service:

“*It*’*s a new word*… *I don*’*t like the term* ‘*intermediate care*’, *I would sit more comfortable with it being a medical rehabilitation ward for older people*.” (HCW22)

“…*the GPs* (general practitioner) *don*’*t know what the heck it is. It*’*s a term that means all things to all men and women and that*’*s the big difficulty*.” (HCW24)

HCWs recognised that the preventative concept of IC was not currently a reality. Despite the definition suggesting that services should prevent unnecessary hospital admissions in addition to providing ‘step-down’ care, IC services were viewed as predominantly providing the latter, catering to patients following a period of care in an acute hospital. The concept of IC being a targeted and time-limited intervention of six weeks was contrasted with a reality of prolonged lengths of stays in IC facilities. HCWs noted that admissions to IC were often inappropriate due to a lack of suitable alternatives and pressures within hospitals to vacate beds:

“*Quite often we would get a lot of social admissions and how appropriate that is I*’*m not quite sure*…*we can be used as a dumping ground*.” (HCW6)

The concept that multidisciplinary working is fundamental to IC was met with the reality that there was no standard approach to the structure of multidisciplinary teams within IC, particularly in relation to medical care provision and pharmacy involvement. Only two of the twelve IC facilities received input from pharmacists and the differences in medical care provision between IC settings are highlighted in Table 2. The pharmacist participants referred to challenges regarding integration into the IC team:

“*When I initially started*, *nursing staff certainly didn*’*t know what my function was*…*they weren*’*t experienced of what a clinical pharmacist would do*…*they couldn*’*t get their head around a pharmacist being able to prescribe or amend kardexes or give them advice*.” (HCW25)

Opinions held by HCWs on the overall concept and effectiveness of the IC service differed depending on the setting of the facility. HCWs within the non-acute hospital IC facilities held positive attitudes towards IC services.

“*I think it*’*s an invaluable service that we are providing. Intermediate care to me is probably the gap between acute service and a patient being allowed to be discharged home* …*I do find it a very very invaluable service really and it*’*s local*, *that means a lot to people as well*.” (HCW15)

However in the more recently established IC models (within nursing and residential homes) opinions differed:

“…*the definition is good*, *the concept is good*…*from the top it looks okay but from the ground it is not running properly*.” (HCW5)

Contrastingly, the majority of patients expressed positive attitudes towards the IC setting and compared services favourably to hospital environments. In particular, they appreciated the homely atmosphere and locality of the facilities and they viewed HCWs as having more time for patient care.

“*I think it*’*s this place that has helped me a lot*…*you just feel like very at home already*.” (P5)

Highlighted in the definition of IC are the concepts of maximising patients’ independence and ‘person-centred care’; however, with regards to medicines management, there was evidence to suggest such principles were not being promoted. Patients were often disempowered with regards to their medicines, due to a distinct absence of self-administration practices and a lack of provision of education to encourage patient independence.

“*There*’*s a fear of letting people*… *take control of their own medication*… *this is an area we still haven*’*t progressed very much from since the conception of the service here*.” (HCW1)

### Setting and supply

The setting of the IC facilities was largely responsible for the primary challenge encountered by some of the facilities, namely the supply of medicines. The nature of the three distinct care settings, as described previously (see Table 2), dictated the medical care provision and hence, the processes surrounding the prescribing and supply of medicines. For instance, non-acute hospital facilities kept supplies of medicines on the premises, unlike the nursing and residential home IC facilities. This, coupled with medical care provision operating ‘off-site’, meant that the process of the supply of medicines to IC patients in such facilities was more logistically challenging.

“…*it*’*s not seamless in any shape or form*…*you are delaying the time from prescription being written to the actual administration of that drug*, *so it*’*s very clumsy*, *the whole management*.” (HCW25)

The challenge of securing a prescriber was a barrier unique to the nursing and residential home settings, where medical care provision and hence, prescribing responsibilities, fell to either the patients’ own GP, a temporary GP local to the IC facility or a Trust-contracted GP referred to as a ‘medical officer’. This variety of prescribing arrangements often resulted in further confusion regarding prescribing responsibilities. HCWs reported on the challenges associated with such fragmented approaches to the prescribing and supply of medicines:

“*It*'*s complicated*…*if the* [patient’s] *GP refused to prescribe scripts then*…*our temporary GP*, *would do it and then he would send it over to this place*…*and then they would send it to the* [hospital pharmacy] *and then we would get the scripts from there. I know*, *it*’*s very complicated*.” (HCW10)

Many HCWs noted that such processes posed safety risks to IC patients:

“…*there has been times we have run out of medication and we just haven*’*t been able to get it quick enough*…*to keep the client*…*on their regular dose*.” (HCW7)

The quantity of medications supplied at discharge at the point of transfer from secondary care to IC was frequently cited as problematic. Often, the supply of medicines from the admitting hospital was insufficient.

“…*diazepam*…*sleeping tablets*, *they*’*ll send you seven tablets* … *those things that are on the PRN* (to be taken when required) *side quite often they don*’*t even send them out*.” (HCW6)

Despite the range of difficulties encountered by HCWs, the majority of patients had no knowledge of who was responsible for prescribing their medicines and had no concerns surrounding their supply.

“*They just give them to me*, *I don*’*t know where they come from*.” (P2)

When HCWs were asked to suggest potential solutions to these barriers, the most frequently suggested solution was to create a supply of medicines on-site, thus bypassing the inherent delays in sourcing medicines from outside the IC setting. The alternative was to ensure an adequate discharge supply of all medicines from the admitting hospital and for community pharmacies to increase their involvement with IC services.

### Responsibility and review

The IC facility setting was the primary factor dictating responsibility for prescribing and review of patients’ medicines. When asked whether medications were reviewed in IC, HCWs noted that this was the responsibility of the doctor. However, in the residential and nursing IC facilities, HCWs reported that doctors visited infrequently, sometimes only when requested by HCWs.

“*We have people who come and don*'*t be seen by the doctor*…*they don*'*t need to see a doctor*.” (HCW7)

There was also an assumption amongst HCWs that patients’ medicines were reviewed in hospital, therefore a review whilst in IC was considered unnecessary. Although most reported not having concerns about their medicines, those patients who wished to have their medications reviewed spoke of the associated difficulties encountered.

“*I*’*d got the chance to talk to* [the doctor]… *I really just wanted off* [the tablet]…*I wasn*’*t impressed*…*I just felt he wasn*’*t taking responsibility*.” (P6)

In all but one facility, patient self-administration of medications was uncommon. Despite this, most HCWs were aware of the relationship between self-administration and independence. However, linking back to the theme of “*concept and reality*”, self-administration evoked patient safety concerns. HCWs reasoned that to retain control and responsibility for medicines administration was a sacrifice in terms of the disempowerment of some patients to ensure safety for all.

“…*it*’*s easier for us to just take control*, *take charge*, *we know they*’*re safely stored*, *we know they*’*ve got them*…” (HCW1)

Both HCWs and patients referred to an expectation amongst patients to be ‘nursed’ when in a healthcare environment, evidenced by the majority of patients readily handing over responsibility of their medicines to staff. However, one patient who had previously managed his own medicines spoke of the resistance met from HCWs when he requested to continue in IC.

“*I think along the lines of* '*that*'*s how we do it here*' *just plain and simple*…*I fairly soon realised it was a fight I wasn*’*t going to win. It was bad*.” (P7)

HCWs viewed the process of assessing patients’ ability to self-administer as time-consuming and incompatible with IC, again highlighting the mismatch between the concept and reality of IC. Responsibility surrounding the required authorisation was also identified as a barrier to self-administration.

“*We have to get the GP to sign that they*’*re happy* [for the patient to self-administer], *but because they come here under a temporary GP*, *Dr M will say* ‘*well I don*’*t know the person*,’ *so he won*’*t sign*.” (HCW12)

Medication counselling describes the practice of providing patients with information about their medicines [13]. This was not considered a responsibility of any HCW and, with the exception of those facilities with pharmacists, would only be initiated at the patients’ request.

“…*if you ask questions*, *you*’*ll be told*, *but you have to enquire instead of being routinely told*.” (P2)

Barriers included a perceived lack of medication knowledge amongst HCWs and views that providing medication counselling was redundant for patients who used medication compliance aids.

“…*patients come in here have maybe been getting blister packs* (a compliance aid) *at home so there*'*s no point*.” (HCW14)

Despite some patients having queries about their medicines, most were apathetic towards the idea of medication counselling.

“*I*'*m one of those people who just takes the doctor*’*s word for it and assume that he knows best and don*'*t really query it*.” (P10)

The contrast in medicines management provision between facilities was most striking when comparing those which had clinical pharmacy involvement and those that did not. The majority of HCWs welcomed the idea of pharmacy integration within the existing multidisciplinary team.

“[Pharmacists] *would be able to*…*look at the kardex and see* ‘*why is this patient on this*? *Do they really need to be on this*?’ …*when I worked in* [hospital] *it was very beneficial*, *the pharmacist*…*done a lot of teaching with the patients*, *even with the staff as well*, *it was great to have somebody there to ask a question if you needed to*.” (HCW18)

Contact with the pharmacy profession amongst most of the facilities was via community pharmacy, with the primary service being the supply of medicines. A minority of HCWs working in facilities without clinical pharmacists did not see a role for the profession within IC beyond the supply of medicines:

“… [Pharmacists’] *input could be only on handling of medication and storage of medications*…*but not really like the MDT*.” (HCW3)

## Discussion

The aims of this study were to describe the current provision of medicines management services in IC facilities in NI and to determine HCWs’ and patients’ views of and attitudes towards these services. This study uncovered disparities between the concept of IC and many aspects of the reality of the services. The setting in which IC was provided was found to be a crucial factor in determining prescribing responsibilities and consequently, arrangements for the supply of medicines to patients. A lack of a standardised approach to the provision of medicines management services including the clinical review of medicines was identified. The lack of integration of pharmacists in IC was evident, and HCWs suggested that increased pharmacy input could offer a potential solution to the identified medicines management deficiencies.

The theme of ‘concept and reality’ symbolised the contrast between the key principles of IC and the service reality. IC is a relatively new concept within the UK, yet it has been integrated into the healthcare system in a variety of models [14]. Community hospitals (non-acute hospital facilities) represent one such model which pre-date the term ‘IC’ but are increasingly being used as IC facilities in the UK [15]. Despite this re-designation, HCWs felt that although IC may be considered a new concept, in reality, the service provided within such facilities remained largely unchanged, providing support to the suggestion that the IC concept is ‘an old idea rebranded’ [16]. Additionally, the confusion surrounding terminology is not unique to NI and has been the subject of previous discussion in the literature [17].

The concept of IC describes services aimed at both preventing unnecessary hospital admissions and providing ‘step-down’ care, however, in NI, the majority of patients are admitted to IC following a period in hospital. The preventative concept of IC is not being realised perhaps due to a lack of awareness of the role of such services within the wider healthcare system. Disengagement with and lack of awareness of IC on the part of both GPs and hospital doctors has been highlighted previously [16, 18]. The unfamiliarity of IC, coupled with pressures faced by acute hospitals to discharge people sooner may contribute to the reported inappropriate admissions to IC in this study. There have been calls to promote awareness among those who refer patients to services, however, evidence is needed to support IC as a care setting [19].

Another concept of IC is the multidisciplinary team. In actuality, this study found that multidisciplinary teams varied considerably between facilities, particularly in relation to medical care provision and pharmacy involvement. If IC is to be a service that seeks to address all aspects of a patient’s care needs including their medicines management needs, a standardised approach to multidisciplinary team working and integration of pharmacists is required. Despite these contrasts between the concept of IC and the reality of the services, patients in this study compared IC favourably to the traditional hospital setting. The perceived benefits of IC for patients were often linked to the ‘home-like’ environment of IC, and have been described previously [20–22].

The theme of ‘setting and supply’ illustrates the impact of the IC setting on medicines management provision and specifically, the supply of medicines. The settings in which IC was delivered within NI were found to be varied, differing not only in the care environment but also the medicines management processes within. The supply of medicines was by far the most frequently voiced medicine-related concern amongst IC staff. Securing a prescription and obtaining medicines from a pharmacy, where a supply was unavailable on-site, presented many difficulties for HCWs. Such gaps in continuity of care have been associated with adverse patient outcomes [23–25]. Conversely, in the non-acute hospital facilities where both prescribers and medicine stock were accessible on-site, such supply issues were practically non-existent. A solution suggested by many HCWs, was to have medication stock available in all IC facilities, thereby eradicating the logistical barriers described. The difficulty with such a proposal would be the requirement for a significant change in the longstanding medication supply arrangements within the nursing and residential homes. Nonetheless, it is evident that the current mode of medication supply within such facilities is not effectively catering for IC patients.

Responsibility for prescribing and reviewing patients’ medicines in the IC facilities varied depending on the setting. There was no standardised approach to the review of patients’ medicines and in the majority of facilities, HCWs noted that medication reviews were not conducted unless explicitly requested. This therefore poses a risk that medication discrepancies and/or ADEs may go undetected amongst older adults in IC facilities. Another barrier to medication review was the assumption of medication optimisation in the acute setting. Caring for older people in residential and nursing home settings is known to have significant workload implications for GPs responsible for their care [26]. The reluctance of GPs to visit patients in institutional settings has been discussed previously and factors such as a desire amongst GPs to maintain strict practice boundaries and the perceived trivial nature of visiting requests have been cited as barriers to the provision of general medical services to older people in these settings [27].

Self-administration of medicines was not promoted in the IC setting. HCWs represented the main barrier to self-administration as they felt the need to be in control of patients’ medicines, citing patient safety as their primary concern. Further resistance to self-administration of medicines came from patients themselves, who were happy to let staff take control of their medicines. There was an expectation among patients of the loss of autonomy when entering a healthcare facility. The same power dynamic between healthcare staff and patients has been described in the traditional nursing home setting [28]. Self-administration of medicines is synonymous with encouraging independence and thus patient empowerment, which itself is associated with improved healthcare outcomes [29]. Specifically, self-administration programmes have been shown to improve compliance with and knowledge of patients' medications post hospital discharge [30, 31]. Despite patients’ indifferent attitudes towards self-administration in this study, it has been demonstrated that following participation in a self-administration programme, the vast majority of patients report a preference for this method over usual nurse administration of medicines [30]. In order to successfully implement self-administration programmes with the view of increasing patient independence, there needs to be a re-examination and acknowledgement of responsibility from not only HCWs but patients as well.

Medication counselling was not routinely provided to patients in IC. A perceived lack of confidence amongst HCWs surrounding knowledge of medications and a view that many older people would not benefit from medication counselling were the two main barriers described. Similar findings have been discussed previously with the result of nurse-patient communications being criticised for often being brief and superficial in nature [32]. Patients’ attitudes proved to be an additional barrier to medication counselling, with many feeling it was simply unnecessary. Medication counselling should be an integral part of patient care, especially in the IC setting where patients are rehabilitating and regaining skills to ensure their independence. Patient education has been shown to improve patients’ ability to remain autonomous with their medications and is frequently a part of interventions aimed at improving adherence to medicines [33]. It has also been associated with a decreased incidence of avoidable adverse drug events post hospital discharge [13], therefore the importance of increasing patients’ understanding of their medicines in the IC setting cannot be underestimated considering that the goal of IC is to promote independence.

Facilitating the supply of medicines, reviewing medicines [34], promoting self-administration [35], and medication counselling [36] are all roles associated with pharmacists. However, it was evident that pharmacists were not integrated members of the IC multidisciplinary team as the pharmacy presence throughout the facilities was minimal. Most facilities referred to community pharmacists as being their sole pharmacy contact who served only to facilitate the supply of medicines. The clinical pharmacists who assumed enhanced pharmacist roles in IC experienced challenges integrating as part of the multidisciplinary team. This may be due to a lack of awareness of the pharmacists’ role, which has previously been identified as a barrier to pharmacist integration in such team [37]. In those facilities without clinical pharmacy input, staff readily identified a range of roles for pharmacists that were currently being unmet. These included medicines reconciliation, liaising with primary and secondary care stakeholders, medicines appropriateness reviews and patient and staff education. Conversely, a minority of HCWs could not see a role for pharmacists in IC, despite identifying several unresolved medicines management challenges. Again, such views may be due to a misunderstanding of the role and stereotypical business orientated image traditionally associated with the profession [38]. There is, therefore, a need for more work to evaluate the role of pharmacists within IC and to raise awareness of the roles of clinical pharmacists.

### Strengths and limitations

This was the first study of its kind to report on the issue of medicines management in the IC setting. As it was qualitative in nature, the results are not intended to be generalizable to all IC facilities. Participation was voluntary, therefore it is possible that the views expressed reflected those with an interest in medicines management, however, data saturation was attained with our sample size and the views described were representative of those held by the majority of the participants. Finally, validation checks were conducted using a second researcher and consensus within the team on the final themes.

## Conclusion

The expected rise in the number and proportion of older people living with chronic conditions and taking multiple medicines will put additional pressure on acute services in the coming decades. IC has been introduced in the UK as one part of the solution to managing this increasing pressure. IC facilities in NI include older established services provided within non-acute hospital settings and more recently, beds within nursing and residential homes. The latter two IC models are fraught with medicines management issues, some of which may pose safety risks to patients. The lack of pharmacy input into the IC setting has been exposed and the potential roles for pharmacists highlighted. IC provides an ideal setting for patients to regain independence and this should also include managing their medicines. Pharmacists are ideally placed to provide advice to both IC staff and patients alike and their skills could be utilised to review medications for appropriateness and help to ensure a seamless transition of care across this healthcare interface. Further work is required to define and evaluate how pharmacists can integrate with IC services.
